# Iron status in ESRD patients

**DOI:** 10.4103/0971-4065.78087

**Published:** 2011

**Authors:** V. Wiwanitkit

**Affiliations:** Wiwanitkit House, Bangkhae, Bangkok, Thailand

Sir,

I read the recent report on iron status in ESRD patients with a great interest.[1] Jairam *et al*. concluded that “our ESRD patients showed marked inflammatory activation, which was more pronounced in patients who had received IV iron.”[1] I agree that the study on the ESRD patients is very interesting. I would like to add some more possible clinical explanations to the observation. Recently, Finkelstein *et al*. studied the correlation between hemoglobin, vitamin C, and peritoneal dialysis.[2] Indeed, the relationship between vitamin C and iron status is confirmed, and vitamin C[3] is also proposed for a possible relationship with hepcidin.[4]
